# Fucoidans as multifunctional marine polysaccharide platforms: From nutritional supplements to advanced drug delivery for cancer therapy

**DOI:** 10.1016/j.ijpx.2026.100546

**Published:** 2026-04-17

**Authors:** Yuning Liu, Lin Long, Jianhua Zang, Chuanlong Guo, Jun Xiao, Gaoyang Lin

**Affiliations:** a Oncology Center I Department, Qingdao Hiser Hospital Affiliated of Qingdao University (Qingdao Traditional Chinese Medicine Hospital), Qingdao 266000, China; b Qingdao Key Laboratory of Biomacromolecular Drug Discovery and Development, College of Chemical Engineering, Qingdao University of Science and Technology, Qingdao 266042, China; c Department of Cardiothoracic Surgery, Qingdao Hiser Hospital Affiliated of Qingdao University (Qingdao Traditional Chinese Medicine Hospital), Qingdao 266000, China

**Keywords:** Fucoidans, Drug delivery systems, Cancer therapy, Nanoparticles, Liposomes, Hydrogels, P-selectin targeting

## Abstract

Fucoidans are sulfated polysaccharides derived from brown algae that have emerged not only as dietary supplements but also as highly promising biomaterials for advanced drug delivery systems, particularly in oncology. This review comprehensively summarizes recent progress in engineering fucoidan-based delivery systems, with a focus on cancer therapy. It highlights their excellent biocompatibility, biodegradability, and intrinsic bioactivities (*e.g.*, antitumor, targeting *via* P-selectin). The design and applications of various fucoidan platforms-including nanoparticles, liposomes, and hydrogels-are detailed, demonstrating their ability to improve drug stability, enable controlled release, and enhance tumor-specific targeting. Key modification strategies, such as thiolation and conjugation with targeting ligands (*e.g.*, anti-epidermal growth factor receptor (EGFR)), are discussed for further optimizing performance. Beyond oncology, the review also covers promising applications in renal protection, wound healing, and antimicrobial therapy. Finally, current challenges such as structural heterogeneity and the gap to clinical translation are addressed, with future directions proposed involving standardization, intelligent design, and comprehensive preclinical validation. This review aims to serve as a strategic reference for the rational design and clinical translation of fucoidan-based nanomedicines.

## Highlights

• Fucoidans are biocompatible marine polysaccharides with antitumor activity and P-selectin targeting for drug delivery.
• This review summarizes fucoidan-based nanoparticles, liposomes, and hydrogels for cancer therapy and modification strategies.
• Current challenges, future perspectives, and applications beyond oncology (*e.g.*, kidney protection) are discussed.

## Graphical abstract

**Figure:**
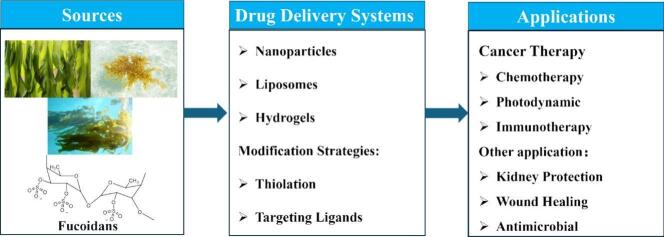

## 1 Introduction

**Fig. 1:**
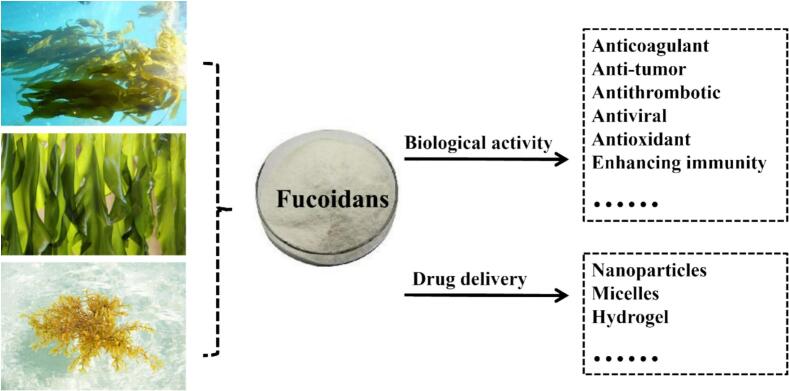
The multifunctionality of fucoidans.

Marine organisms account for a significant proportion of global biodiversity, with numerous species producing bioactive metabolites. Many marine organisms, such as *green algae*, *red algae* and *brown algae*, produce metabolites with unique chemical structures, which have a wide range of biological activities (Pereira, 2020). Fucoidans refer to a class of sulfated polysaccharides rich in L-fucose, which is predominantly extracted from the cell walls of brown algae (Wang et al., 2020). Fucoidans exhibit a variety of pharmacological activities, including anti-tumor, antioxidant, immunomodulatory, lipid-lowering, anticoagulant, and antiviral effects (Alekseyenko et al., 2007; Hayashi et al., 2008; Silva et al., 2005; Tissot et al., 2003). Fucoidans exhibit a broad spectrum of pharmacological activities, underscoring their considerable potential not only as dietary supplements but also as therapeutic agents. The biological activities of fucoidans have attracted considerable attention (Fig. 1). For instance, their anti-hypertensive, anti-diabetic, anti-obesity, and lipid-lowering effects have been reported in both preclinical studies and clinical trials (Villar et al., 2022). In addition, they also deserve attention for their antioxidant, anti-inflammatory, immune regulation, anti-tumor, anticoagulant, antibacterial, and neuroprotective effects (Jin et al., 2022; Villar et al., 2022; Wang et al., 2022; Zhang et al., 2022). More and more evidence also shows that fucoidans can prevent a variety of kidney problems, including acute kidney injury, renal fibrosis and diabetes nephropathy (Liu et al., 2021; Yu et al., 2020). Fucoidans reduce the accumulation of extracellular matrix and slow down renal fibrosis and glomerulosclerosis, indicating their potential in the development of drugs for treating kidney diseases (Zahan et al., 2022).

In recent years, a large number of studies have focused on the development of nano-drug delivery system based on natural active polysaccharide-fucoidan (Liu et al., 2022; Venkatesan et al., 2022). The molecular chains of fucoidans contain various functional groups, primarily sulfate groups, along with hydroxyl groups and, depending on the source, carboxyl groups. These structural features, together with their multi-branched architecture, provide multiple modifiable sites for chemical functionalization. Purposeful chemical modification of fucoidans can modify their physicochemical properties, enabling their use in drug delivery systems to improve the solubility, bioavailability, efficacy, and targeting of therapeutic agents (Venkatesan et al., 2022). The integration of fucoidans with nanotechnology offers synergistic advantages, positioning fucoidan-based systems as promising tools in biomedical applications (Zvyagintseva et al., 2021).

This review aims to provide a comprehensive and critical analysis of the development of fucoidan-based drug delivery systems, with a particular emphasis on their application in oncology. The biocompatibility of fucoidans, design strategies for various nanocarriers (nanoparticles, liposomes, and hydrogels), and key functionalization approaches to enhance targeting and controlled release are systematically explored. Furthermore, promising applications beyond cancer, including in renal protection, wound healing, and antimicrobial therapy, are discussed. Finally, current challenges and future perspectives are addressed, aiming to offer valuable insights for the rational design of next-generation fucoidan-enabled therapeutics.

## 2 Chemical and physical properties of fucoidans

Fucoidans represent a class of heterogeneous sulfated polysaccharides with L-fucose as the core monosaccharide unit. Their structural diversity, chemical composition, and physical properties are highly dependent on their algal sources, extraction and purification methods, and even the growth stage and environmental conditions of the original algae (Kiselevskiy et al., 2022; Venkatesan et al., 2022). This structural and property heterogeneity forms the core basis for their diverse biological activities and the design of functionalized drug delivery systems. In this section, this review systematically classifies fucoidans based on their source characteristics, and clarify the core chemical and physical properties of different types of fucoidans and their structure–activity relationship basis.

### 2.1 Classification and sources of fucoidans

Fucoidans are mainly extracted from the cell wall and intercellular matrix of brown algae (Phaeophyceae), with smaller amounts isolated from some marine invertebrates (*e.g.*, sea cucumbers, sea urchins) (Jayawardena et al., 2022; Zahan et al., 2022). According to the main brown algal source genera, fucoidans can be divided into several major types with distinct structural characteristics, each possessing specific physicochemical properties and biological activity tendencies (Table 1).

**Table 1:** Classification, sources and specific properties of major fucoidan types.

Fucoidan Type	Main Sources	Core Structural Characteristics	Key Physical/Chemical Properties	References
Fucus-derived	*Fucus vesiculosus*, *Fucus evanescens*	α-(1 → 3)-L-fucose backbone, SD = 20–35%, C2/C4 sulfation, MW = 10–100 kDa	High sulfation, good P-selectin binding affinity, moderate water solubility	(Anastyuk et al., 2012; Bilan et al., 2002; Flórez-Fernández et al., 2023)
Undaria-derived	Undaria pinnatifida sporophyll	α-(1 → 3)/(1 → 4)-L-fucose hybrid backbone, SD = 15–25%, C2 sulfation, MW = 5–40 kDa	High water solubility, low cytotoxicity, good oral bioavailability	(Synytsya et al., 2010; Yu et al., 2021)
Laminaria-derived	Laminaria japonica, *Laminaria digitata*	α-(1 → 3)-L-fucose backbone, SD = 10–20%, MW = 50–200 kDa	High MW, multi-branched, strong gel-forming ability, good tissue adhesion	(Li et al., 2024; Wang et al., 2010)
Sargassum-derived	Sargassum wightii, Sargassum siliquosum	α-(1 → 3)/(1 → 4)-L-fucose hybrid backbone, SD = 18–30%, high rhamnose content, MW = 15–80 kDa	Good thermal stability, strong antibacterial activity, pH stability	(Puhari et al., 2022; Wang et al., 2020)
Invertebrate-derived	*Apostichopus japonicus* (sea cucumber)	α-(1 → 3)-L-fucose backbone, amino sugar modification, SD = 10–15%	Low anticoagulant activity, strong cell adhesion inhibition, moderate water solubility	(Yu et al., 2015)

Note: SD = Sulfation Degree; MW = Molecular Weight.

### 2.2 Core chemical and physical properties of fucoidans

Regardless of the source, fucoidans share a set of core chemical and physical properties that form the basis for their application as nutritional supplements and drug delivery carriers (Haggag et al., 2023). Combined with the specificity of different types, the key physicochemical properties are summarized as follows:

#### 2.2.1 Sulfation characteristics

Sulfate groups (-O-SO_3_^−^) are the most important functional groups of fucoidans, serving as the core determinants of their biological activity and physicochemical behavior (Chen et al., 2018; Ren et al., 2019). The sulfation degree (SD) of fucoidans ranges from 10% to 35% across different types, and the sulfation sites (C2, C4, C6 of fucose) also show source specificity (Table 1). Sulfate groups endow fucoidans with a negative surface charge (zeta potential usually −20 to −70 mV) (Ostolska and Wiśniewska, 2014), which is the basis for their electrostatic assembly with positively charged materials (*e.g.*, chitosan, PEI) to form nanoparticles, and also the key for their specific binding to P-selectin (a positively charged transmembrane glycoprotein overexpressed on tumor vasculature) (Ho et al., 2022). For example, *Fucus*-derived fucoidans with high SD have stronger P-selectin binding affinity, making them more suitable for tumor-targeted drug delivery.

#### 2.2.2 Molecular weight and solubility

The molecular weight of fucoidans ranges from 5 kDa (oligo-fucoidan) to 200 kDa (macromolecular fucoidan), and their water solubility is negatively correlated with molecular weight and positively correlated with sulfation degree (Tran et al., 2021). Oligo-fucoidans (MW < 10 kDa) from *Undaria pinnatifida* have excellent water solubility and oral bioavailability (Nagamine et al., 2014), making them suitable for oral drug delivery systems, while macromolecular fucoidans (MW > 50 kDa) from *Laminaria japonica* have poor solubility but strong gel-forming ability (Wang et al., 2010), which is suitable for hydrogel preparation. In addition, degradation of fucoidans (*e.g.*, enzymatic hydrolysis, ultrasonic degradation) can adjust their molecular weight, thereby optimizing their solubility and biological activity for specific drug delivery needs (Lu et al., 2017).

#### 2.2.3 Functional groups and modifiability

The molecular chains of fucoidans contain a variety of reactive functional groups, including sulfate groups, carboxyl groups (often derived from uronic acid residues), and hydroxyl groups (Yu et al., 2015). Of note, some fucoidans, particularly those isolated from certain brown algae, have been reported to contain small amounts of amino sugars (Nishino et al., 1994). These groups provide multiple modifiable chemical sites for fucoidans, enabling chemical modifications such as thiolation, esterification, and conjugation with targeting ligands (*e.g.*, anti-EGFR, TPP). For example, the hydroxyl group at C-6 of fucose can be thiolated to form FUC-SH, which can self-assemble with doxorubicin to form pH/GSH dual-responsive nanoparticles (Qiongyan et al., 2025); the reactive groups on fucoidan can be utilized to conjugate with tumor-targeting ligands, such as anti-EGFR antibodies, to enhance the targeting ability of fucoidan-based delivery systems (Shanmugapriya et al., 2020). The modifiability of fucoidans is the core reason they can be flexibly engineered into various drug delivery platforms (nanoparticles, liposomes, hydrogels).

#### 2.2.4 Colloidal stability and biodegradability

Fucoidans have good colloidal stability in aqueous solution due to their negative charge and steric hindrance from multi-branched chains (Ostolska and Wiśniewska, 2014), which can prevent the aggregation of nanoparticles/liposomes in physiological environments. Meanwhile, fucoidans are natural polysaccharides that can be biodegraded by glycosidases (*e.g.*, fucosidase) in the human body, and their degradation products are L-fucose and inorganic sulfate, which are non-toxic and can be excreted through the renal system (Kim et al., 2010). This biodegradability and biosafety ensure that fucoidan-based drug delivery systems have no long-term toxic side effects *in vivo*, representing a key advantage compared with synthetic polymer carriers.

#### 2.2.5 Thermal and pH stability

Fucoidans have good thermal stability (denaturation temperature > 100 °C) and pH stability in the physiological pH range (pH 5.0–7.4). *Sargassum*-derived fucoidans can maintain their structural integrity at 80 °C for 24 h (Ramu et al., 2020), and *Undaria*-derived fucoidans can remain stable in the acidic environment of the stomach (pH 1.2–3.0) and release drugs in the intestinal tract (pH 6.0–7.0) (Nagamine et al., 2014). This stability makes fucoidans suitable for the preparation of oral drug delivery systems and *in vivo* sustained release systems.

The above chemical and physical properties not only form the basis for the diverse biological activities of fucoidans but also serve as key design principles for fucoidan-based drug delivery systems. In the subsequent chapters, the specific properties of different fucoidan types are further correlated with the design of delivery systems to clarify the rationale for selecting specific fucoidan types for different therapeutic applications.

## 3 Biocompatibility and antitumor potential of fucoidans

Fucoidans, sulfated polysaccharides primarily composed of L-fucose, are recognized not only for their excellent biocompatibility and safety profile but also for their inherent, multi-faceted antitumor properties, which form a critical foundation for their application in oncology.

### 3.1 Biosafety and metabolic profile

The safety of fucoidans is well-established through extensive *in vitro* and *in vivo* studies. Acute and subacute toxicity tests in rodents have shown no significant adverse effects following oral administration (Ramu et al., 2020). Furthermore, clinical observations in human subjects have confirmed their safety (Kim et al., 2010). Pharmacokinetic studies indicate that after oral intake, fucoidans were absorbed through the intestinal tract, exhibit preferential accumulation in organs such as the kidneys, liver, and spleen, and were ultimately cleared *via* renal excretion (Nagamine et al., 2014; Shang et al., 2018). This favorable biosafety and metabolic profile ensure a reliable foundation for their therapeutic use.

### 3.2 Direct antitumor activity and modulation of the tumor microenvironment

Fucoidans exert direct antitumor effects through multiple mechanisms, including inducing apoptosis, inhibiting tumor cell proliferation and metastasis, and suppressing tumor-associated angiogenesis (Gao et al., 2025; Jia et al., 2025; Uyen et al., 2025). Concurrently, they actively remodels the tumor microenvironment by modulating immune responses-such as activating B cells, T cells, and NK cells-and reducing chronic inflammation (Amer et al., 2025; Guo et al., 2022b). Notably, their high-affinity binding to P-selectin, commonly overexpressed on tumor vasculature and metastatic sites, provides an intrinsic targeting capability (Ho et al., 2022; Tylawsky et al., 2023; Wang et al., 2021). This dual role, combining direct cytotoxicity with microenvironment regulation, positions fucoidans as unique “active carriers” that can synergize with encapsulated therapeutics to enhance overall antitumor efficacy (Kiselevskiy et al., 2022).

## 4 Fucoidans used as drug delivery carriers for cancer treatment

### 4.1 Fucoidan-based nanoparticles

Drug delivery systems (DDS) can deliver drugs to specific positions of human tissues, control the release rate of drugs, improve the specificity and bioavailability of drugs, avoid damage to normal tissues, and improve the therapeutic effect of lesions (Chourasia and Jain, 2003). Among them, the choice of a drug delivery carrier is the most important. The molecular chain of fucoidans contains numerous active groups, such as sulfate and carboxyl groups, which facilitate chemical modifications and the formation of nanocomplexes (Zvyagintseva et al., 2021). Hydrogen bonds in fucoidans are also important factors for the formation of stable and ordered nanocomplexes (Wijesinghe and Jeon, 2012). Fucoidans possess excellent biological activity, safety and degradability, which proves their great application prospect in drug delivery systems (Nagamine et al., 2014; Shang et al., 2018).

**Fig. 2:**
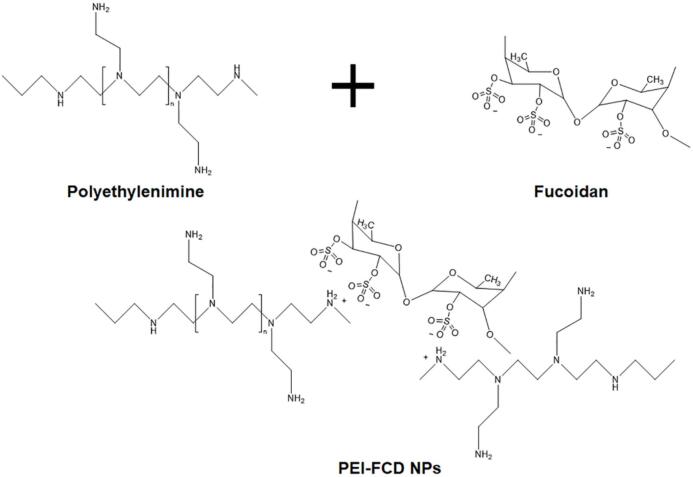
Preparation of fucoidan-polyvinyl imide composite nanoparticles (PEI-FCD NPs) (Pawar et al., 2019). Copyright © 2019, Elsevier.

In aqueous systems, negative or positive charges in nanoparticles contribute to their repulsion and prevent them from aggregating and becoming unstable (Ostolska and Wiśniewska, 2014). Pawar et al. assembled fucoidan (FCD) with positively charged polyethyleneimine (PEI) electrostatically, which was negatively charged and the particle size ranged from 41 nm to 160 nm (Pawar et al., 2019). The fucoidan-polyvinyl imide composite nanoparticles (PEI-FCD NPs) were prepared by dispersing them in mannitol solution (5% *w*/*v*) and gelatinizing overnight (Fig. 2). Notably, the PEI-FCD NPs loaded with doxorubicin exhibited enhanced anticancer activity in a 4 T1 breast cancer model by promoting immunogenic cell death and activating antitumor immune responses, demonstrating their potential for chemo-immunotherapy. Choi et al. dissolved chitosan (CS) with 0.25% (*v*/*w*) Tween-80 in 1% (*v*/v) acetic acid solution. Then, fucoidan was dissolved in deionized water, and added to chitosan solution with stirring to form chitosan-fucoidan composite nanoparticles (Choi et al., 2019). These nanoparticles were used to deliver pro-oxidant drugs, showing selective cytotoxicity toward cancer cells while sparing normal cells, highlighting their potential for targeted cancer therapy.

**Fig. 3:**
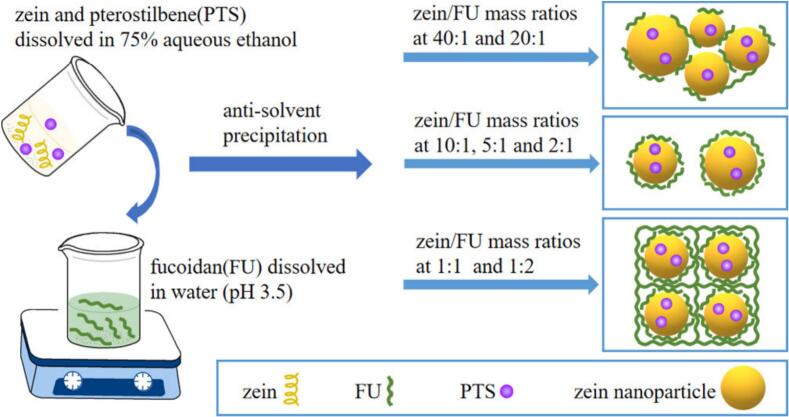
Preparation of pterostilbene-zein/fucoidan nanoparticles (Liu et al., 2020). Copyright © 2020, Elsevier.

Liu et al. prepared pterostilbene-loaded zein/fucoidan composite nanoparticles (zein/FU-PTS), which are driven by electrostatic force, hydrogen bonding, and hydrophobic interactions (Liu et al., 2020). Briefly, zein (1%, w/v) and pterostilbene (PTS) (0.1%, w/v) were dissolved in 75% (*v/v*) ethanol aqueous solution, and fucoidan was added to deionized water (pH adjusted to 3.5 with 1 M HCl) and dissolved with stirring. Then, the zein/PTS mixture was slowly added into the fucoidan solution under continued stirring, and ethanol was removed by rotary evaporation to obtain pterostilbene-loaded zein/fucoidan nanoparticles with an average diameter of 120–150 nm (Fig. 3). The resulting nanoparticles significantly enhanced the stability and bioavailability of pterostilbene, a natural anticancer agent, and exhibited improved antiproliferative activity against cancer cells, suggesting their potential as an effective oral delivery system for cancer therapy.

**Fig. 4:**
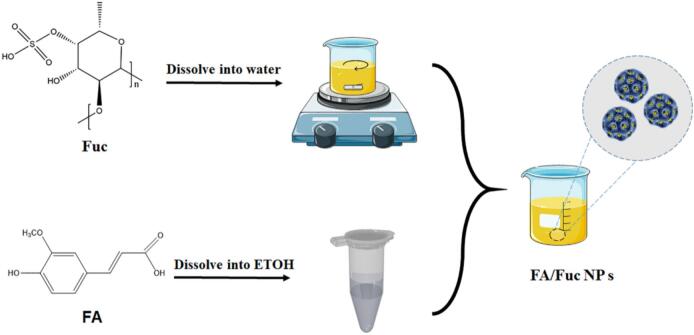
Preparation of FA/FU nanoparticles (FA/FU NPs). Copyright © 2022, Elsevier.

Anti-solvent method utilizes the different solubility of drugs in two different solvents to prepare nanoparticles, which has the advantages of simplicity and mild conditions. Gao et al. prepared fucoidan-ferulic acid nanoparticles (FA/FU NPs) by anti-solvent method (Gao et al., 2022). The fucoidan was dissolved in deionized water and stirred for 2 h. Ferulic acid was dissolved in absolute ethanol to obtain a uniform solution. Under stirring, the ferulic acid-ethanol solution was added dropwise into the fucoidan aqueous solution, followed by continued stirring to evaporate the ethanol, yielding water-dispersed FA/FU nanoparticles (FA/FU NPs) (Fig. 4). Although this study focused on the protective effects against cisplatin-induced acute kidney injury, the established nanoparticle formulation also holds promise for cancer therapy, as ferulic acid has been reported to exhibit antitumor activity and fucoidan itself possesses P-selectin targeting capability.

Lai et al. prepared fucoidan-docetaxel nanoparticles by an emulsion-solvent evaporation method. Specifically, poly(lactic-*co*-glycolic acid) (PLGA) and docetaxel (DTX) were dissolved in chloroform, mixed with 2 mL of 0.5% (*w*/*v*) fucoidan solution, and the chloroform was removed after sonication (10 W, 26 kHz) to obtain FPN-DTX nanoparticles (Lai et al., 2020). *In vivo* studies demonstrated that FPN-DTX nanoparticles significantly inhibited tumor growth in a prostate cancer xenograft model compared to free DTX, with improved pharmacokinetics and reduced systemic toxicity; underscoring the potential of fucoidan-based nanocarriers for enhanced chemotherapeutic delivery. Chiang et al. used an emulsification method to mix the aqueous phase containing fucoidan with the dichloromethane organic phase containing PLGA and soybean oil, followed by sonication. After the single-phase emulsion was formed, the sample was placed in a rotary evaporator to remove the organic phase to obtain fucoidan-PLGA nanoparticles (Chiang et al., 2021). These inherently therapeutic fucoidan-PLGA nanoparticles exhibited anticancer activity without drug loading, which was attributed to the intrinsic bioactivity of fucoidan, offering a unique strategy for cancer treatment with minimal side effects.

**Fig. 5:**
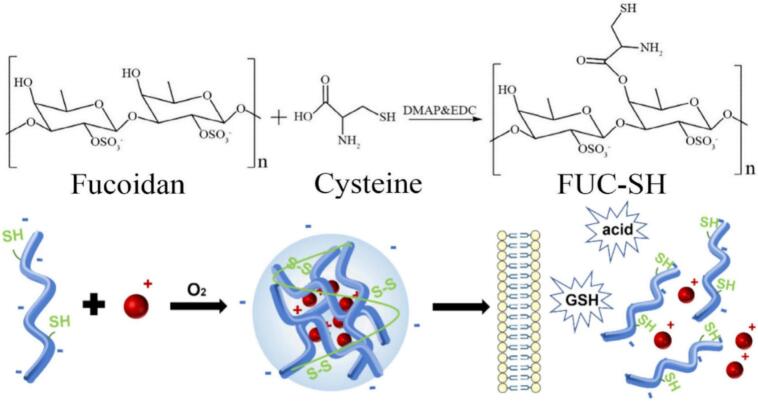
Thiolated fucoidan (FUC-SH) and doxorubicin (DOX) were self-assembled into nanoparticles exhibiting pH/GSH dual-responsive drug release (Qiongyan et al., 2025). Copyright © 2025, Elsevier.

Fucoidan–chitosan composite nanoparticles are a research hotspot. The combination of chitosan's biocompatibility and mucoadhesive properties with the targeting ability of fucoidans significantly improves drug delivery efficiency. Additionally, composite nanoparticles formed from fucoidans, and protamine offer a new option for delivering peptide and protein drugs. Recent studies have also developed nanoparticles formed by self-assembly of thiolated fucoidan (FUC-SH) and doxorubicin (DOX), which exhibit pH/GSH dual-responsive drug release (Fig. 5). These nanoparticles enable selective drug release in the tumor microenvironment, with a drug loading efficiency as high as 70.97 ± 1.70% (Qiongyan et al., 2025). Such stimuli-responsive systems are particularly advantageous for cancer therapy, as they allow controlled release of chemotherapeutics specifically within tumor cells, thereby enhancing efficacy while reducing off-target toxicity.

**Fig. 6:**
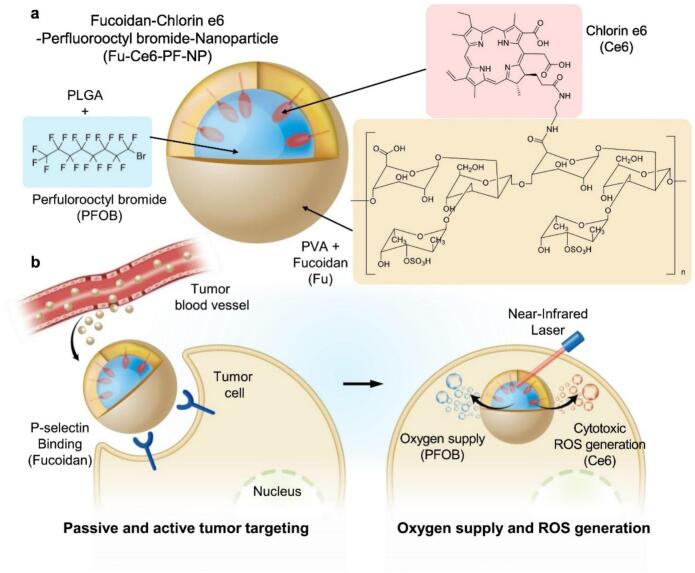
Schematic illustration of perfluorooctylbromide (PFOB)-loaded fucoidan-chlorin e6 nanoparticles (Fu-Ce6-PF-NPs) and their therapeutic application. (a) Structure of Fu-Ce6-PF-NPs. (b) Strategy for tumor-targeted and enhanced photodynamic therapy (Han et al., 2022). Copyright © 2022, Elsevier.

Guo et al. studied the development of fucoidan functionalized micelles (FD/DOX), which can effectively adhere to activated platelets by targeting P-selectin and increase its distribution in tumor tissues and metastases (Mahalingam et al., 2014). The developed nanoparticles had a particle size of 120 nm and a zeta potential of 20 mV, which showed excellent anti-tumor and anti-metastasis effects on 4 T1 spontaneous metastasis model. In addition, the experiment also verified that FD/DOX nanoparticles inhibited the expression of transforming growth factor-b (TGF-b), thus stimulating anti-tumor immune response and reversing immunosuppressive microenvironment. Han et al. prepared fucoidan (Fu)-dihydroporphyrin e6 (Ce6) nanoparticles (NPs) containing perfluorooctane bromide (PFOB) by combining fucoidan (extracted from brown algae) with hydrophobic Ce6 (Fig. 6) (Han et al., 2022). The cytotoxic reactive oxygen species produced by Ce6 killed tumor cells under laser irradiation. Compared with free Ce6, the fluorescence intensity of Ce6 treated with Fu-Ce6-PF-NP was 4.70 times higher than that treated with free Ce6. PF can supply oxygen to hypoxic tumor tissues and improve the curative effect of photodynamic therapy (PDT). Fu-Ce 6-PF-NP has the potential to be used as a tumor-targeted drug carrier for PDT therapy. Due to the binding of P-selectin and rapid uptake of cells into SCC 7 tumor cells, Fu-Ce6-PF-NP group produces a large amount of ROS based on strong fluorescence signal, which is 4.26 times and 1.96 times higher than that of free Ce6 and Fu-Ce6-NP irradiated by laser, respectively. Nanoparticles can effectively release oxygen, resist hypoxia and kill tumor cells after laser irradiation. In SCC7 tumor-bearing mice, after intravenous injection of passive and active tumor targeting, the circulation time of NP in blood was longer and the accumulation in tumor tissue was higher. After laser irradiation, PDT treated NP has obvious anti-tumor effect. It is proved that Fu-Ce6-PF-NP is a promising tumor-targeting drug carrier that binds to p-selectin.

Lu et al. prepared a pH-and enzyme-responsive nano-carrier using protamine and fucoidan as carriers for DOX delivery (Lu et al., 2017). The pH/enzyme response characteristics were verified by circular dichroism (CD), dynamic light scattering (DLS) and zeta potential analysis. The nanoparticles induced the release of adriamycin in enzymatic digestion and the intracellular acidic microenvironment (pH 4.5–5.5). The protamine/fucoidan nanoparticles have P-selectin-mediated endocytosis, charge conversion and stimulation-adjustable release properties. The survival rate of breast cancer metastatic cell line (MDA-MB-231) treated with protamine/fucoidan nanoparticles (2.5 μg/mL DOX content) decreased significantly to 44.5% after 48 h incubation. Hwang et al. designed fucoidan-cisplatin nanoparticles (FCNPs) by mixing cisplatin with fucoidan solution (Hwang et al., 2017). The particle size was 181.2 ± 21.0 nm, and the zeta potential was −67.4 ± 2.3 mV. The protective effect of FCNPs on RAW264.7 cells was verified, which showed potential application value in immunotherapy and chemotherapy.

Tumor hypoxia is the main mechanism of drug resistance induced by radiotherapy, and is associated with poor prognosis in tumor patients (Oh et al., 2026). Shin et al. prepared fucoidan coated manganese dioxide nanoparticles (Fuco-MnO_2_-NPs) and found that Fuco-MnO_2_-NPs reversed hypoxia induced radiation resistance, reduced clone formation, increased DNA damage, and cell apoptosis responsive to radiotherapy (Shin et al., 2018). Fuco-MnO_2_-NPs also reduced the expression of HIF-1α in pancreatic cancer cells cultured under hypoxia, indicating that a large amount of oxygen is produced. Consistent with *in vitro* data, the combination of Fuco-MnO_2_-NPs and radiation significantly inhibited the growth of BxPC-3 xenograft tumor *in vivo*. Intratumoral injection of Fuco-MnO_2_-NPs also alleviated tumor hypoxia *in vivo* by targeting HIF-1α.

Guo et al. designed and prepared a novel reactive oxygen species (ROS)-responsive nano-drug based on fucoidan by conjugating an aggregation-induced emission (AIE) molecule, tetraphenylethylene (TPE), to the caffeic acid (CA)-modified fucoidan (FUC) amphiphilic carrier material (termed CA-FUC-TK-TPE, CFTT). The study also utilized a thioketal (TK)-linked vitamin E (VE) and fucoidan amphiphilic carrier material (FUC-TK-VE, FTVE) (Guo et al., 2022a). Paclitaxel (PTX) and Fe^3+^ nanoparticles (CT/PTX) were formed by self-assembly of CFTT and FTVE in water. The size of CT/PTX nanoparticles was 150.3 ± 12.0 nm with a polydispersity index (PDI) of 0.098 ± 0.029. CT/PTX induced ROS oxidative stress at tumor sites through Fe^3+^-induced chemodynamic therapy (CDT). *In vitro* release tests demonstrated ROS sensitivity. ROS detection assays showed that CT/PTX nanoparticles could generate a ROS cascade after entering tumor cells. *In vivo* and *in vitro* studies demonstrated that CT/PTX nanoparticles could reduce toxic side effects, enhance tumor accumulation, and improve anticancer activity.

Catarina et al. prepared fucoidan/chitosan nanoparticles functionalized with ErbB-2 antibody on the surface and used them for gemcitabine loading (Oliveira et al., 2021). The maximum immobilization of ErbB-2 on the surface of nanoparticles was 10 μg/mL, the particle size was 160 nm, with a PDI of 0.18, and the zeta potential was 21 mV. The targeting ability was verified by comparing the uptake of MDA-MB-231 (ErbB-2 negative) and SKBR 3 cells (ErbB-2 positive), and the composite nanoparticles significantly reduced tumor growth and lung metastasis.

Samar et al. synthesized novel fucoidan nanoparticles (FCD/LF NP) by interacting negatively charged fucoidan with positively charged active targeting ligand lactoferrin (Etman et al., 2020). The particle size was 167 nm and the zeta potential was −27 mv. The IC_50_ for pancreatic cancer cells was close to 20 μg/mL. The FCD/LF NP nanosystem showed enhanced ability to prevent the migration and invasion of pancreatic cancer cells. Chiang et al. prepared an intrinsic therapeutic fucoidan-glucan magnetic nano-drug (IO@FuDex3) combining checkpoint inhibitor (anti-PD-L1) and T cell activator (anti-CD3 and anti-CD28) (Chiang et al., 2018). The coincidence nanoparticles directly regulate the immune system to inhibit the tumor microenvironment. Composite nanoparticles exhibit better safety compared to soluble anti PD-L1 antibodies, extending the median survival time from 32 days to 63 days.

### 4.2 Liposome delivery system

Liposomes are classical drug delivery carriers known for their good biocompatibility and ability to encapsulate both hydrophilic and hydrophobic drugs. However, they suffer from limitations such as poor stability and insufficient targeting. Fucoidans modification can effectively address these drawbacks. By coating or encapsulating fucoidans on the liposome surface, a targeted and highly stable liposomal delivery system can be prepared (Lima Salviano et al., 2021; Obiedallah et al., 2024).

**Fig. 7:**
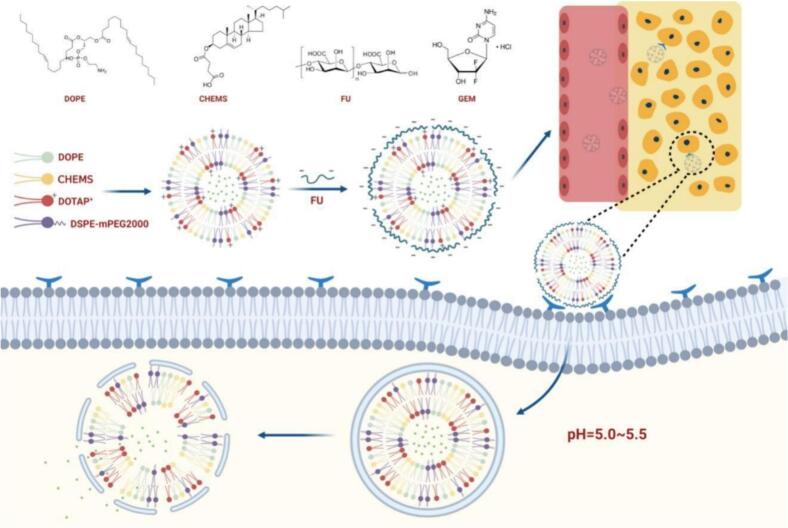
Designed for pH-triggered release, fucoidan-coated liposomes deliver gemcitabine with stability under physiological conditions and rapid drug liberation in the acidic niche of pancreatic tumors (Zheng et al., 2024). Copyright © 2024, Elsevier.

Fucoidan-coated liposomes are a common modification strategy (Han et al., 2026; Han et al., 2025). The sulfate groups in fucoidans can bind to amino groups on the liposome surface to form a stable coating, which not only enhances the circulatory stability of liposomes *in vivo* but also enables targeted delivery through interactions between fucoidans and specific receptors on cell surfaces (Liu et al., 2023; Wu et al., 2024). For example, fucoidan-coated pH-sensitive liposomes delivering gemcitabine remain stable under normal physiological pH but rapidly release the drug upon entering the acidic microenvironment of pancreatic cancer tissue (Fig. 7) (Silli et al., 2024; Zheng et al., 2024). To enhance the bioavailability of phytol, Chen et.al developed zein/fucoidan-coated nanoliposomes *via* magnetic stirring combined with high-pressure homogenization. The resulting nanoparticles exhibited high encapsulation efficiency, excellent physicochemical stability, and sustained release under simulated gastrointestinal conditions, along with significantly improved antioxidant activity. These findings demonstrate that the zein/fucoidan coating serves as an effective delivery system for hydrophobic natural products, enhancing their stability and bioactivity (Chen et al., 2024). Simultaneously, fucoidans target tumor cells, significantly increasing drug concentration at tumor sites, enhancing inhibitory effects, and reducing systemic side effects. Moreover, fucoidan-modified liposomes can deliver photosensitizers, chemotherapeutic drugs, and other agents, showing promising applications in photodynamic therapy and chemotherapy for tumors (Cruz-Lugo et al., 2025; Xu et al., 2019).

### 4.3 Hydrogel delivery system

Hydrogels are hydrophilic polymeric materials with three-dimensional network structures capable of absorbing large amounts of water, making them suitable as carriers for local or sustained drug release. When combined with other polymers or crosslinkers, fucoidans can contribute to the formation of hydrogel delivery systems, which are particularly suitable for local tumor therapy, wound healing, and similar applications (Shanmugapriya and Kang, 2021; Zheng et al., 2025). Fucoidan-based hydrogels can be categorized into those in which fucoidans serve as the primary structural component and composite hydrogels. Hydrogels with fucoidans as the primary structural component can be prepared *via* metal ion crosslinking or chemical crosslinking, although such systems still require the addition of crosslinking agents. Composite hydrogels combine fucoidans with materials such as chitin, chitosan, and hyaluronic acid (HA) to optimize performance. For instance, fucoidan–chitin hydrogels combine the bioactivity of fucoidans with the mechanical strength of chitin, making them suitable as tissue engineering scaffolds and drug delivery carriers (Afrin et al., 2026; Qureshi et al., 2025; Shanmugapriya and Kang, 2021).

**Fig. 8:**
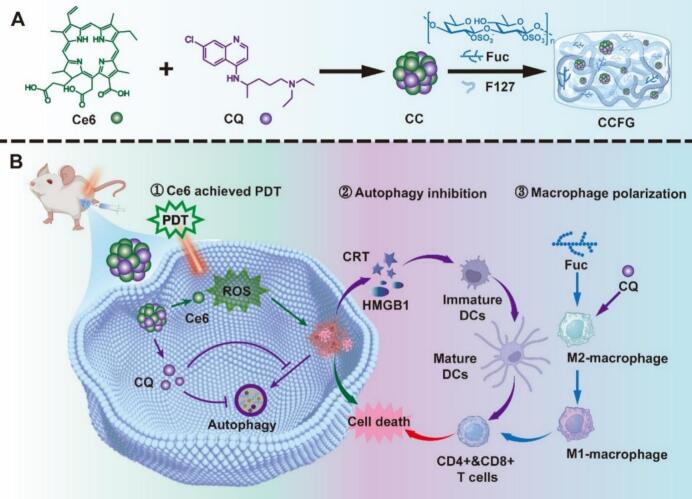
Schematic illustration of CCFG functioning as an *in situ* vaccine. (A) Preparation of CCFG. (B) The three-step mechanism of CCFG-mediated tumor inhibition: (Wang et al., 2024). Copyright © 2024, Elsevier.

In recent years, injectable and self-healing fucoidan hydrogels have become a research focus (Afrin et al., 2026; Zhang et al., 2025). Imine-crosslinked injectable self-healing fucoidan hydrogels exhibit good biocompatibility and injectability, allowing minimally invasive administration (Liu et al., 2026; Yin et al., 2025). They rapidly gelate *in vivo* and possess self-healing capability, adapting to irregular shapes of tissue defects (Qureshi et al., 2025). Fucoidan–Ce6–chloroquine hydrogels combine photodynamic therapy, chemotherapy, and immunotherapy, achieving synergistic effects through sustained drug release and significantly improving tumor treatment outcomes (Fig. 8) (Wang et al., 2024). Additionally, injectable hydrogels formed *via* Michael addition between thiolated fucoidan and polydopamine (PDA) can encapsulate drug-loaded nanoparticles, enabling combined chemo-photothermal therapy and showing excellent tumor suppression in breast cancer models (Gao et al., 2024).

## 5 Modification strategies of fucoidans and their impact on delivery performance

To further enhance the performance of fucoidan-based delivery systems, researchers have modified the structure of fucoidans through chemical strategies, primarily including the following two approaches:

### 5.1 Thiolation modification

**Fig. 9:**
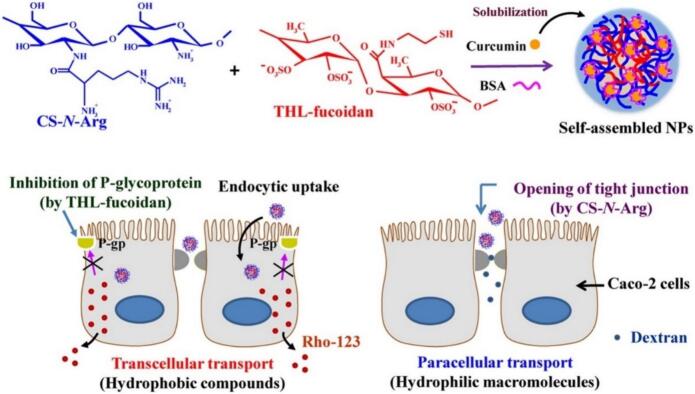
Multifunctional nanoparticles based on arginine-modified chitosan and thiolated fucoidan for oral co-delivery of hydrophobic and hydrophilic drugs (Chen et al., 2018). Copyright © 2018, Elsevier.

Chien-Ho Chen et.al developed self-assembled nanoparticles from arginine-modified chitosan and thiolated fucoidan to enhance intestinal delivery. The NPs exhibited pH-sensitive drug release, disrupted epithelial tight junctions, and inhibited P-glycoprotein, thereby significantly improving the paracellular permeation of macromolecules and the cellular uptake of hydrophobic curcumin (Fig. 9) (Chen et al., 2018).

### 5.2 Targeting molecule modification

**Fig. 10:**
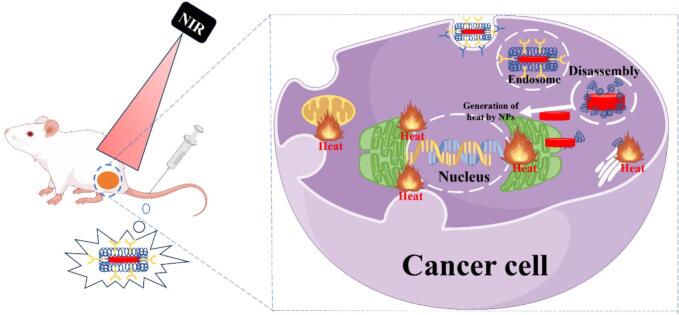
Fucoidan-Coated Gold Nanorods Conjugated with Anti-EGFR Antibody as a Novel Photothermal Ablation Platform for Cancer Therapy (Manivasagan et al., 2017). Copyright © 2017, American Chemical Society. (For interpretation of the references to colour in this figure legend, the reader is referred to the web version of this article.)

Conjugating fucoidans with tumor-specific targeting molecules, such as antibodies or peptides, can significantly enhance the targeting capability of the delivery system. While fucoidans can bind to receptors overexpressed on tumor cell surfaces (*e.g.*, P-selectin, EGFR) to achieve a certain degree of targeting, further modification with targeting molecules greatly improves tumor cell recognition. For instance, EGFR-conjugated fucoidan/alginate hydrogels target colon cancer cells *via* the EGFR/AKT signaling pathway, demonstrating excellent targeting efficacy in photodynamic therapy (Shanmugapriya et al., 2020). Fucoidan-coated and anti-EGFR antibody-conjugated gold nanorods have emerged as promising photothermal ablation agents for cancer nanotheranostics. These nanoparticles, with an average size of 96.37 ± 3.73 nm, combine excellent biocompatibility, target specificity, and strong near-infrared absorption, making them effective for targeted photothermal therapy under 808 nm laser irradiation at 2 W/cm^2^ (Fig. 10) (Manivasagan et al., 2017).

Xue et.al developed a dual-targeted nanodelivery system (FU@TPP/PTE Mn^2+^ NPs) for breast cancer treatment by combining P-selectin-targeting fucoidan and mitochondrial-targeting TPP to co-deliver pterostilbene and Mn^2+^. The system effectively induced mitochondrial apoptosis and activated the cGAS-STING immune pathway, leading to significant tumor growth inhibition, reduced metastasis, and enhanced immune cell activation in a 4 T1 breast cancer model. These findings demonstrate the promising therapeutic potential of simultaneously targeting mitochondria and modulating the immune pathway for advanced breast cancer therapy (Xue et al., 2026).

## 6 The role of fucoidans in other delivery systems

### 6.1 Fucoidan nanoparticles for drug delivery of kidney diseases

**Fig. 11:**
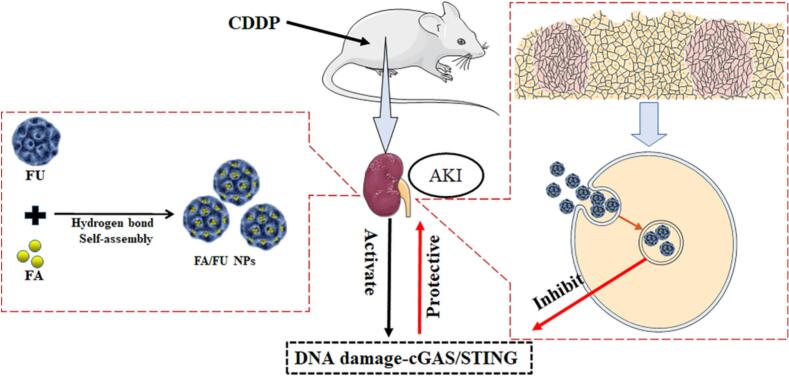
Kidney protective effects of FA/FU NPs. Copyright © 2022, Elsevier.

Fucoidans can modulate pathological risk factors of a variety of kidney diseases, including inflammation, oxidative stress and fibrosis, thus preventing age-related kidney diseases, including AKI and CKD (Kwon et al., 2017; Sohn et al., 2017; Uddin et al., 2021). Gao et al. prepared ferulic acid (FA) fucoidan nanoparticles (FA/FU NPs) with a particle size of 158.6 ± 4.5 nm, and the renal protective mechanism was preliminarily discussed (Fig. 11). *In vitro* experiments showed that FA/FU NPs significantly inhibited cisplatin (CDDP)-induced HK-2 cell apoptosis, ROS accumulation and MMP reduction, reverse CDDP-induced inhibition of cell proliferation, and inhibit CDDP-induced DNA damage by inhibiting cGAS-STING pathway. In addition, *in vivo* results confirmed the protective effect of FA/FU NPs on CDDP-induced acute kidney injury (AKI). Compared with cisplatin group, the renal tubular injury score decreased by 59.79%. In urea nitrogen level, compared with CDDP group, FA/FU NP group decreased by 67.95%. Compared with CDDP group, the creatinine level in FA/FU NPs group decreased by 48.88%. Further studies have confirmed that FA/FU NPs play a protective role in kidney by changing the activities of various antioxidant enzymes in kidney tissue (Gao et al., 2022).

Shin et al. developed an antioxidant and anti-inflammatory polymer prodrug PVU 73 that can be activated by H_2_O_2_, using a peroxyoxalic acid bond that is easily cleaved by H_2_O_2_ to covalently bind vanillin (VA) and ursodeoxycholic acid (UDCA), and coated it with fucoidan to form Fu-PVU 73 nanoparticles. Fu-PVU 73 nanoparticles can be targeted by the adhesion molecule P-selectin overexpressed on endothelial cells at the inflammatory site. They release UDCA and VA during H₂O₂ degradation, inhibiting the excessive production of intracellular ROS and the overexpression of pro-inflammatory cytokines. The *in vitro* model validated that Fu-PVU 73 nanoparticles can effectively clear H_2_O_2_ and protect cells from H_2_O_2_ mediated cytotoxicity. In an *in vivo* ischemia reperfusion (IR) renal injury model, Fu-PVU 73 nanoparticles accumulated in the kidney, significantly reducing renal injury and creatinine and urea nitrogen levels, inhibiting ROS production, TNF and IL-1 expression, and cell apoptosis (Shin et al., 2022).

Wang et al. developed a dual-targeted nano-delivery system (Fu-4-PBA/Po NPs) that combines P-selectin-targeting fucoidan, the endoplasmic reticulum (ER) stress inhibitor 4-PBA, and the natural antioxidant polydatin. The nanoparticles exhibited sustained release, enhanced pharmacokinetics, and effectively alleviated oxidative stress and ER stress by inhibiting the PERK–eIF2α–ATF4–CHOP and cGAS–STING pathways. *In vivo*, they significantly improved renal function and histopathology, demonstrating strong potential for the clinical treatment of cisplatin-induced acute kidney injury (AKI) (Wang et al., 2025).

### 6.2 Antimicrobial activity of fucoidan-based nanoparticles

Khan et al. used fucoidan as a stabilizer and reductant to synthesize fucoidan gold nanoparticles in chlorogold acid solution, and the particle size ranged from 15 nm to 19 nm (Khan et al., 2019). The minimal inhibitory concentration (MIC) of F-AuNPs was 512 μg/mL. The minimal biofilm inhibitory concentration (MBIC) and minimal biofilm eradication concentration (MBEC) of F-AuNPs against *Pseudomonas aeruginosa* were determined to be 128 μg/mL due to its stable and water-soluble characteristics. High concentration of F-AuNPS (512 μg/mL) could kill bacteria, while sub-MIC F-AuNPS could inhibit the formation of biofilm and eradicate mature, established biofilm. F-AuNPS sub-MIC also inhibited the production of pyocyanin and rhamnolipid by *Pseudomonas aeruginosa*. Under different doses of fucoidan-coated gold nanoparticles, the inhibition rates of pyocyanin and rhamnolipid were 79.4%, 81.9% and 87.7%, and 54%, 50% and 53%, respectively, and weakened the clustering, swimming and convulsive motility of bacteria. F-AuNPS inhibited the hemolytic activity of *Pseudomonas aeruginosa* in a concentration-dependent manner.

Vijayakumar et al. prepared fucoidan coated gold nanoparticles (Fu-AuNPs), which are spherical to triangular crystals with a particle size of 10–100 nm and a zeta potential of −23.5 mV. At 100 μg/mL, the antimicrobial range of the synthesized FU-Au NPs against *Aeromonas hydrophila* was 23.2 mM, higher than that of commercial antibiotic chloramphenicol (17.3 mM), and the MIC of FU-Au NPs (1.875 μg/mL) was lower than that of bare chloroauric acid (HAuCl4) and of fucoidan crude extract (Vijayakumar et al., 2017). Fu-Aunps had higher inhibitory activity against gram-negative (*Aeromonas hydrophila*) biofilm. The results of light microscope and confocal laser scanning microscope showed that 100 μg/mL Fu-Au NPs completely blocked the biofilm of hydrophilic pathogen. Cytotoxicity studies showed that Fu-Au NPS effectively inhibited the activity of human cervical cancer cells (HeLa) at the concentration of 100 μg/mL, and induced cell morphological changes. In another experiment, the antibacterial effect of Fu-Au NPs was evaluated *in vivo* in *Oreochromis mossambicus* infected with *Aeromonas hydrophila*. The mortality rate of infected fish was 90%, whereas treatment with Fu-Au NPs reduced the mortality rate to 30%.

Ravichandran et al. synthesized fucoidan containing silver nanoparticles (Fu-Ag NPs), and formed spherical nanoparticles with size of 74.56 nm, and zeta potential of −23.5 mV (Ravichandran et al., 2018). Silver nanoparticles can interact with DNA and hinder the replication process of bacteria (Wirth et al., 2016). The results of disk diffusion test showed that Fu-Ag NPS inhibited the growth of *Streptococcus pneumoniae* (*Klebsiella pneumoniae*), exhibiting stronger antibacterial activity than fucoidan alone.

Fernandes-Negreiros et al. synthesized silver nanoparticles (FN) containing fucoidan (Fernandes-Negreiros et al., 2017). FN was spherical and negatively charged within 16 months, with an average size of 103.3 ± 43 nm. FN (1 mg/mL) inhibited the proliferation of melanoma cell line B16 F10 (60%). When FN was used at a concentration of 2 mg/mL, only 80% of human osteosarcoma cells were inhibited. FN also had immune regulatory properties, causing up to 7000 times of nitric oxide and cytokines (IL-10, IL-6 and TNF-α) levels. In addition, FN inhibited both Gram-positive (*E. coli*) and Gram-negative (*S. golden yellow*) bacteria with a minimum inhibitory concentration (MIC) of 50 μg/mL. In general, FN was proved to be an anti-tumor agent, immunomodulator and antibacterial agent. Previous studies have proved the antibacterial effect of silver nanoparticles (Chen et al., 2016; Venkatpurwar and Pokharkar, 2011; Wirth et al., 2016). Ag NP exhibited antimicrobial activity at higher concentrations (0.8 to 1.6 mg/mL) than those used to achieve the same effect with FN.

### 6.3 Fucoidan-based gels for wound dressing

Tannic acid (TA) was mixed with tilapia skin gelatin (Gel) and fucoidan (Fuc) to prepare a gelatin-fucoidan-tannic acid (Gel-Fuc-TA) wound dressing hydrogel with good network structure and swelling release characteristics by the TA impregnation method (Lu et al., 2022). The polymers were cross-linked by non-covalent hydrogen bonds to form a three-dimensional porous structure, which improved the thermal stability of Gel-Fuc-TA hydrogel polymer. The swelling properties of the gel and the slow-release properties of Fuc and TA were verified. Compared with the untreated group, Gel-Fuc-TA hydrogel treatment significantly reduced the blood loss from 513 mg and 430 mg to 186 mg and 130 mg respectively (*P* < 0.01), showing obvious hemostatic effect. In the model of wound therapy, Gel-Fuc-TA hydrogel regulated the diversity of wound microorganisms, reduced the colonization of wound microorganisms, and the expression of pro-inflammatory factors (such as lipopolysaccharide (LPS), Toll-like receptor 2 (TLR2) and Toll-like receptor 4 (TLR4)) related to wound microbial community through the antibacterial effect of TA. Gel-Fuc-TA hydrogel also promoted the expression of vascular endothelial growth factor (VEGF), platelet endothelial cell adhesion molecule-1 (CD-31), α-smooth muscle actin (α-SMA), enhanced collagen deposition and accelerated wound repair. It showed excellent antibacterial, antioxidant, cell compatibility and hemostatic properties. The Gel-Fuc-TA hydrogel showed great prospect as a wound dressing.

Yu et al. developed a microneedle array patch (KCFMN) using Kangfuxin (KFX), chitosan (CS) and fucoidan (FD), which was conical, with a head end diameter of 5 μm, a height of 700 μm and a bottom diameter of 300 μm (Yu et al., 2022). KCFMN patch could penetrate the stratum corneum of the skin, promoting the penetration of KFX and marine polysaccharide, and overcoming the problem of low percutaneous penetration rate of traditional drugs. The antibacterial activity of KCFMN patch against *Staphylococcus aureus* was detected. The results showed that compared with the control group, the number of colonies in KCFMN and CFMN groups decreased significantly, and the inhibition rate of KCFMN against *E.coli* was close to 100%, and against *S. aureus* was 80%. *In vivo* experiments confirmed that KCFMN patch significantly promoted wound healing by improving the thickness of epithelium and collagen deposition.

### 6.4 Regulation of atherosclerosis

Fucoidans can bind to type I and II transmembrane glycoprotein receptors found on the surface of macrophages. These receptors, also known as macrophage scavenger receptors (MSR), are modulators of protein kinase signal transduction pathway, which can regulate inflammatory cytokines TNF and IL-1 related to atherosclerosis formation (Hsu et al., 2001). Lira et al. prepared fucoidan (isocyanoacrylic acid) nanoparticles by anionic emulsion polymerization (AEP) and redox radical emulsion polymerization (RREP) of isobutyl cyanoacrylate. Compared with non-macrophages, fucoidan incorporated on the surface of nanoparticles could promote the interaction between nanoparticles and macrophages, showing great potential to regulate the formation of atherosclerosis (Lira et al., 2011).

Fucoidans are sulfated polysaccharides with anticoagulant and antithrombotic properties, which has been proved to have potential effects on endothelial cell adhesion and proliferation (Min et al., 2012). Yao et al. used the adjustable mechanical properties and low thrombotic surface properties of a polyvinyl alcohol (PVA) hydrogel and modified it by blending fucoidan with polyvinyl alcohol, which were co-crosslinked by sodium tripolyphosphate (STMP), to obtain a fucoidan-polyvinyl alcohol gel (PVA-F). The average density of fucoidan was 53.4 ± 2.0 μg/cm^2^ by quantitative determination (Yao et al., 2020). The adhesion ability of HUVEC on PVA, PVA-F and glass coverslip was analyzed. It was verified that the presence of fucoidan on PVA significantly improved the adhesion and coverage of endothelial cells on PVA, while maintaining the blood compatibility of PVA. The patency rate of PVA-F grafts in rabbits was 80%. Immunostaining revealed increased endothelialization (CD31 and eNOS) and decreased intimal hyperplasia (α-SMA). In addition, PVA-F exhibited low platelet adhesion and activation, as well as reduced thrombin production. These studies showed the potential of PVA-F for its wide application in developing vascular implants and instruments.

## 7 Current challenges and future perspectives of fucoidan-based drug delivery systems

Fucoidans are marine sulfated polysaccharides with excellent biocompatibility, intrinsic bioactivities, and structural modifiability. They have been engineered into three core drug delivery systems—nanoparticles, liposomes, and hydrogels—for oncology and other therapeutic fields, each with distinct clinical applicability. Nanoparticles enable hydrophilic/hydrophobic drug co-delivery, active targeting, and stimuli-responsive release, making them ideal for systemic tumor combination therapy. However, they suffer from batch inconsistency due to fucoidan heterogeneity and large-scale production hurdles. Fucoidan-modified liposomes offer enhanced *in vivo* stability and tumor targeting, making them suitable for systemic long-circulation chemotherapy, but are limited by drug-dependent encapsulation efficiency and storage-related oxidation or drug leakage. Hydrogels support local sustained release with injectable and self-healing properties for local tumor therapy and wound healing, yet face challenges such as low mechanical strength and limited applicability for systemic administration. Their key characteristics are summarized in Table 2, and future design strategies can integrate their complementary strengths (*e.g.*, hydrogel “nano-reservoirs”) to address tumor heterogeneity and metastasis.

**Table 2:** Comparative overview of fucoidan-based drug delivery systems: nanoparticles, liposomes, and hydrogels.

System Type	Key Advantages	Main Limitations	Typical Applications	Representative Examples in Text
Nanoparticles	• Diverse preparation methods (self-assembly, emulsion, anti-solvent, *etc.*) • Controllable size and ease of functionalization • Co-delivery capability for both hydrophilic and hydrophobic drugs • Enables active targeting (*e.g.*, *via* P-selectin) and stimuli-responsive release (pH/enzyme/ROS)	• Structural heterogeneity may affect batch-to-batch consistency • Long-term stability in physiological environments requires further improvement for some formulations • Challenges in process standardization for large-scale production	• Systemic administration (*e.g.*, intravenous) for tumor-targeted therapy • Combination therapies: chemotherapy, photodynamic therapy, immunotherapy	• PEI-FCD NPs (Pawar et al., 2019) • Thiolated FUC-SH/DOX NPs (Qiongyan et al., 2025) • Fu-Ce6-PF-NPs (Han et al., 2022)
Liposomes	• Excellent biocompatibility and biodegradability • Well-established formulation technology • Fucoidan coating significantly enhances stability and targeting • Amenable to stimuli-responsive (*e.g.*, pH-triggered) drug release	• Unmodified liposomes are susceptible to clearance by the mononuclear phagocyte system • Potential issues during long-term storage: phospholipid oxidation or drug leakage • Drug encapsulation efficiency highly dependent on drug properties	• Systemic chemotherapy requiring long circulation and tumor targeting • Encapsulation of photosensitizers for targeted photodynamic therapy	• Fucoidan-coated, pH-sensitive gemcitabine liposomes (Zheng et al., 2024) • Zein/fucoidan-coated nanoliposomes (Chen et al., 2024)
Hydrogels	• High water content and excellent biocompatibility • Ideal for local injection or implantation enabling sustained drug release • Injectable and self-healing formulations adapt to irregular lesion sites • Serves as a multifunctional platform to combine therapies (chemotherapy/phototherapy/immunomodulation)	• Generally exhibit lower mechanical strength • Not suitable for systemic administration; primarily for local treatment • Precise control over gelation kinetics and drug release profiles can be complex	• Local tumor therapy (*e.g.*, post-surgical residual disease) • Wound dressings and tissue engineering • Local immunomodulatory microenvironment remodeling	• CCFG *in situ* vaccine hydrogel (Wang et al., 2024) • Gel–Fuc–TA wound dressing (Lu et al., 2022)

Despite notable progress, fucoidan-based delivery systems face three interrelated challenges hindering clinical translation, with structural heterogeneity as the fundamental bottleneck. Fucoidans' composition and sulfation features vary with algal source and extraction methods, causing raw material inconsistency and poor preclinical reproducibility due to a lack of unified quality standards. Many formulations also exhibit insufficient physiological stability: nanoparticles aggregate and release drugs prematurely *in vivo*, hydrogels degrade rapidly in inflammatory microenvironments, and liposomes suffer storage oxidation or drug leakage. Most critically, a stark preclinical–clinical gap exists, with most systems remaining in *in vitro* or *in vivo* stages, hampered by difficulty in scalable production, inadequate biosafety evaluation (lack of long-term large-animal data), and unoptimized dosing regimens.

Future research should focus on standardizing fucoidan extraction and purification. It must also clarify the structure–property relationships of these compounds. Researchers should engineer fucoidan structures through chemical modification, combined with artificial intelligence, to develop smart nanomedicines. Another priority is optimizing scalable, GMP-compliant production *via* interdisciplinary collaboration. Finally, preclinical and clinical studies need to be advanced to validate efficacy, expand applications, and explore combination therapies.

## 8 Conclusion and prospect

In summary, fucoidans represent distinctive and beneficial seaweed-derived polysaccharides that serve as effective drug delivery platforms through various physicochemical interactions. This review has examined their applications as nanocarriers in biomedicine, exemplified by their utility in delivering doxorubicin for cancer treatment and ferulic acid for AKI recovery. Moving forward, refining the fabrication of fucoidan-based nanoparticles will be key to bridging the gap between preclinical studies and clinical practice. As clinical trials progress, fucoidans are poised to transition into commercial entities, thereby promoting the sustainable development of the blue bioeconomy.

## CRediT authorship contribution statement

**Yuning Liu:** Writing – original draft, Software, Investigation. **Lin Long:** Writing – original draft, Investigation. **Jianhua Zang:** Writing – original draft, Investigation. **Chuanlong Guo:** Writing – review & editing, Software, Investigation. **Jun Xiao:** Writing – review & editing, Funding acquisition. **Gaoyang Lin:** Writing – review & editing, Investigation.

## Declaration of competing interest

The authors declare that they have no known competing financial interests or personal relationships that could have appeared to influence the work reported in this paper.

## Data Availability

No data was used for the research described in the article.
